# Prognostic Implication of Lymphovascular Invasion in Early Gastric Cancer Meeting Endoscopic Submucosal Dissection Criteria: Insights from Radical Surgery Outcomes

**DOI:** 10.3390/cancers16050979

**Published:** 2024-02-28

**Authors:** Bo Sun, Huanhuan Li, Xiaodong Gu, Hong Cai

**Affiliations:** 1 Department of Gastric Surgery, Fudan University Shanghai Cancer Center, Shanghai 200032, China; 15111220073@fudan.edu.cn; 2 Department of Oncology, Shanghai Medical College of Fudan University, Shanghai 200032, China; zl_huanhuanli@fudan.edu.cn; 3 Department of Radiology, Fudan University Shanghai Cancer Center, Shanghai 200032, China; 4 Department of General Surgery, Huashan Hospital, Fudan University, Shanghai 200031, China

**Keywords:** early gastric cancer, endoscopic submucosal dissection, lymphovascular invasion, lymph node metastasis, prognostic factors

## Abstract

**Simple Summary:**

Early gastric cancer is a condition that can often be treated effectively if caught early. A less invasive treatment, known as endoscopic submucosal dissection (ESD), has become more popular for these early-stage cancers. However, deciding which patients are best suited for this treatment requires more information. This study looked into how the presence of cancer cells in the lymph or blood vessels, a condition called lymphovascular invasion (LVI), affects the chance of cancer returning or spreading in patients eligible for ESD. By reviewing the medical records of 1369 patients treated in Shanghai, we discovered that LVI greatly increases the risk of cancer spreading to the lymph nodes and negatively affects patients’ survival rates. Our findings suggest that patients with LVI may need more aggressive treatment, such as surgery, to remove the cancer completely. This information could help doctors better decide on treatment plans for patients with early gastric cancer, potentially improving their chances of a successful recovery. This research highlights the importance of considering LVI in the treatment of early gastric cancer, which could lead to changes in how these patients are cared for in the future.

**Abstract:**

Background: The management of early gastric cancer (EGC) has witnessed a rise in the utilization of endoscopic submucosal dissection (ESD) as a treatment modality, although prognostic markers are needed to guide management strategies. This study investigates the prognostic implications of lymphovascular invasion (LVI) in ESD-eligible EGC patients, specifically its implications for subsequent radical surgery. Material and methods: A retrospective, multicenter study from two primary hospitals analyzed clinicopathological data from 1369 EGC patients eligible for ESD, who underwent gastrectomy at Shanghai Cancer Center and Huashan Hospital between 2009 and 2018. We evaluated the relationship between LVI and lymph node metastasis (LNM), as well as the influence of LVI on recurrence-free survival (RFS) and overall survival (OS). Results: We found a strong association between LVI and LNM (*p* < 0.001). Advanced machine learning approaches, including Random Forest, Gradient Boosting Machine, and eXtreme Gradient Boosting, confirmed the pivotal role of LVI in forecasting LNM from both centers. Multivariate analysis identified LVI as an independent negative prognostic factor for both RFS and OS, with hazard ratios of 4.5 (95% CI: 2.4–8.5, *p* < 0.001) and 4.4 (95% CI: 2.1–8.9, *p* < 0.001), respectively. Conclusions: LVI is crucial for risk stratification in ESD-eligible EGC patients, underscoring the necessity for radical gastrectomy. Future research should explore the potential incorporation of LVI status into existing TNM staging systems and novel therapeutic strategies.

## 1. Introduction

Early gastric cancer (EGC) is characterized by the confinement of tumor invasion to the mucosa or submucosa layers of the stomach wall, regardless of whether regional lymph node metastasis (LNM) is present or not [1]. Early detection and treatment significantly improve patient prognosis, emphasizing the critical need for effective interventions for EGC [2]. Endoscopic submucosal dissection (ESD) has emerged as a favored treatment strategy for EGC [3]. This method is less invasive than conventional surgeries, offering additional benefits like enhanced quality of life, the preservation of organs, and economic efficiency. Yet, ESD is not a universal solution for all EGC patients. Authoritative bodies, including the Japanese Gastric Cancer Association (JGCA), European Society for Medical Oncology (ESMO), Korean Gastric Cancer Association (KGCA), and National Comprehensive Cancer Network (NCCN) have issued clinical practice guidelines for ESD, increasingly refining the distinction between patients suited for ESD versus radical gastrectomy [4,5,6,7].

Lymphovascular invasion (LVI), the infiltration of neoplastic cells into the lymphatic or blood vessels, presents an escalated risk for cancer metastasis [8,9]. This pathological feature is a key determinant in prognosticating various cancer types [10,11]. Several studies have identified LVI as an independent risk factor for LNM and a predictor of a poor prognosis in EGC patients [12,13]. The JGCA and KGCA guidelines advocate for supplemental surgery in EGC patients with positive LVI. The ESMO guidelines refer to the European Society of Gastrointestinal Endoscopy, which aligns with the Japanese criteria regarding LVI. Similarly, the NCCN guidelines acknowledge the significance of LVI, highlighting the necessity for thorough investigation into its prognostic implications.

In this study, we specifically focus on patients who underwent radical gastrectomy, selecting a subset that meets the current indications for ESD, to evaluate the prognostic value of LVI. By doing so, we aim to discern the impact of LVI on surgical decision making and assess its implications for patients initially considered suitable for less invasive ESD. This approach allows us to investigate the pathological characteristics that indicate ESD suitability and to explore how LVI influences critical outcomes such as recurrence-free survival (RFS) and overall survival (OS). Our objective is to provide substantial evidence that will contribute to the ongoing debate regarding the need for additional surgery in patients post-ESD, particularly those with positive LVI findings.

## 2. Material and Methods

### 2.1. Study Population

**Figure 1:**
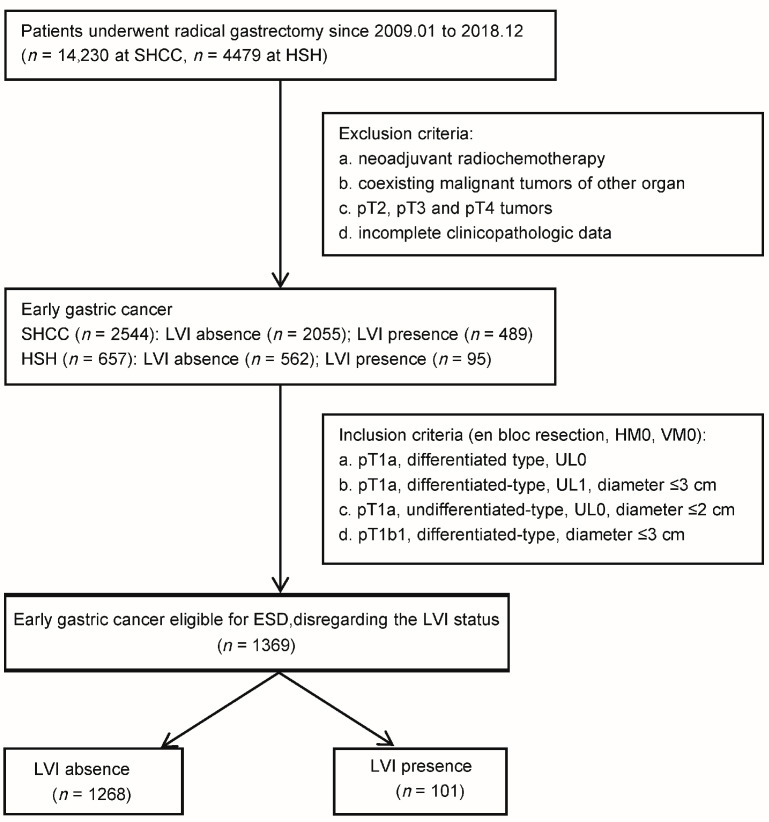
Flowchart of patients with early gastric cancer meeting ESD Criteria. LVI, lymphovascular invasion; ESD, endoscopic submucosal dissection; UL, ulcer; SHCC, Shanghai Cancer Center; HSH, Huashan Hospital.

We conducted a multicenter retrospective cohort study, where we collected data from patients who underwent radical gastrectomy for gastric cancer across two major cancer centers from January 2009 to December 2018 (Figure 1). From an initial 14,230 cases from Shanghai Cance Center (SHCC) and 4479 cases from Huashan Hospital (HSH), we made exclusions for patients who had received neoadjuvant therapy, underwent combined cancer surgeries, or had incomplete clinicopathologic data. This led to a refined cohort comprising 3201 EGC patients, including 2544 from the Shanghai Cancer Center (SHCC) and 657 from Huashan Hospital (HSH). For the purpose of this specific analysis, we further narrowed our focus to EGC patients who were eligible for ESD, based on both the ‘absolute’ and ‘expanded’ criteria from the Japanese gastric cancer treatment guidelines 2021 (6th edition), disregarding their LVI status [6]. These were as follows: (a) pT1a differentiated-type adenocarcinomas without ulcerative findings (UL0), independent of tumor diameter. (b) pT1a differentiated-type adenocarcinomas with ulcerative findings (UL1) and a diameter ≤ 3 cm. (c) pT1a undifferentiated-type adenocarcinomas without ulcerative findings (UL0) with a diameter ≤ 2 cm. Alongside these criteria, we included patients with pT1b EGC classified under endoscopic curability B (eCuraB) who met all the following conditions: en bloc resection, histologically differentiated type-dominant, pT1b1 (SM1) (<500 μm from the muscularis mucosae), HM0, VM0, and a tumor size ≤ 3 cm [6]. Ultimately, our investigation focused on a total of 1369 ESD-eligible EGC patients, comprising 1045 from SHCC and 324 from HSH.

### 2.2. Data Collection

Information was meticulously extracted from each patient’s record, which encompassed demographic variables, specifically age and gender. A detailed analysis of pathological features, which included tumor type, size, depth of invasion, ulcerative findings, histological grade, lymph node involvement, surgical margin status, nerve invasion status, and LVI status, was rigorously conducted. Additionally, surgical intervention details, such as the type and duration of the surgery and any postoperative complications, were scrupulously documented. To ensure the accuracy and reliability of the extracted data, two seasoned clinicians independently reviewed a representative sample of records. Furthermore, it was documented whether patients fulfilled the endoscopic submucosal dissection (ESD) eligibility criteria and adhered to the endoscopic curability A and B (eCuraA and eCuraB) criteria. All patient data were anonymized prior to analysis to uphold patient confidentiality.

The primary outcome measures or dependent variables in our study are OS and RFS. OS refers to the interval commencing from the surgical intervention date to either the date of death regardless of the cause, or the latest follow-up. RFS represents the timespan from the date of surgery until the onset of first recurrence, fatality attributable to gastric cancer, or the final follow-up. For postoperative monitoring, patients undergo a physical examination, serum tumor marker tests, chest X-ray, and abdominal–pelvic CT scan every three months in the first two years. From the third to the fifth year, evaluations are conducted biannually, and thereafter, patients are assessed annually. Additionally, a gastroscopy is performed yearly. Positron emission tomography (PET)-CT and magnetic resonance imaging (MRI) were utilized for diagnostic clarification if initial evaluations were inconclusive. Patient follow-up adhered to consistent, standard protocols, ensuring uniform assessment intervals and methodologies throughout the study duration.

### 2.3. Statistical Analysis

All statistical analyses in this study were conducted using SPSS version 22.0 (IBM Corp., Armonk, NY, USA). Categorical variables were reported as frequencies and percentages, whereas continuous variables were denoted as either means with standard deviation (SD) or medians accompanied by interquartile ranges (IQR), as deemed appropriate. The Chi-square or Fisher’s exact test examined the variances between groups in categorical variables for univariate analysis, while Student’s *t*-test or the Mann–Whitney U test was used for continuous variables, as suitable.

To ascertain the predictive value of diverse factors for LNM in EGC patients, we harnessed an intricate machine learning approach, encompassing three leading algorithms: Random Forest (RF), Gradient Boosting Machine (GBM), and eXtreme Gradient Boosting (XGBoost) [14,15]. Efficacy was meticulously assessed using an array of performance indicators, namely accuracy, Area Under the Curve (AUC), sensitivity, specificity, precision, F1 score, and the Kappa statistic.

OS and RFS rates estimation employed the Kaplan–Meier method, with the log-rank test used for survival curves comparison. Significant variables in the univariate analysis were incorporated into the multivariate analysis to pinpoint independent risk factors for OS and RFS. For multivariate analysis, the Cox proportional hazards regression model was used, following a forward stepwise method. The findings of the Cox regression analysis were delineated as hazard ratios (HRs) with 95% confidence intervals (CIs). A *p*-value less than 0.05 (two-sided) was deemed statistically significant. A biostatistician supervised all data handling and statistical analyses to ensure result accuracy and dependability.

## 3. Results

### 3.1. Clinicopathological Characteristics of EGC Patients Meeting ESD Criteria

In our analysis of EGC patients meeting the ESD criteria, the mean age showed no significant difference based on LVI status (*p* = 0.629) (Table 1). A predominance of male patients was observed, with a significant impact of LVI status (*p* < 0.001). Tumor differentiation exhibited higher undifferentiated tumor prevalence in LVI-positive patients (*p* = 0.081). Tumor depth was significantly associated with LVI status (*p* < 0.001). Neither tumor size nor ulcer presence associated significantly with LVI status. Nerve invasion, although a rare event, was significantly correlated with LVI (*p* < 0.001). LNM was also significantly associated with LVI (*p* < 0.001), emphasizing its prognostic importance in ESD-eligible EGC patients. Specifically, the LNM rate was 1.2%, but this escalated to 43.6% in LVI-positive patients. This finding established the significant role of LVI in predicting LNM.

In our cohort, 49.2% of the patients were treated with D1 or D1+ dissection, whereas 50.8% underwent D2 dissection. A variety of reconstruction techniques were applied. Billroth-I reconstruction was the most common method, utilized in 31.6% of cases. Billroth-II and the modified Billroth-II with Braun anastomosis were employed in 10.1% and 19.9% of patients, respectively. Roux-en-Y reconstruction was used in two contexts: after distal gastrectomy (14.5%) and after total gastrectomy (12.9%). Esophagogastrostomy after proximal gastrectomy was performed in 7.5% of cases, while a double-tract reconstruction after proximal gastrectomy was used in 3.4% of patients.

### 3.2. Evaluating LVI as a Predictive Marker for LNM

**Figure 2:**
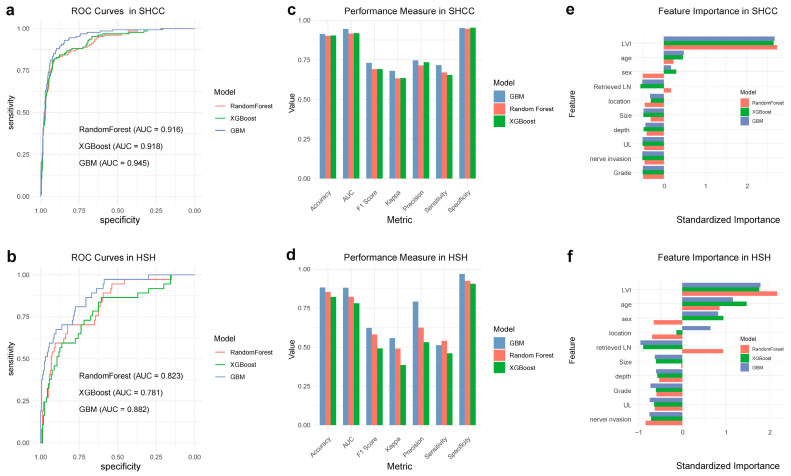
Comparative performance of predictive algorithms for assessing the prognostic significance of LVI in predicting LNM within EGC patients across two centers. (**a**,**b**) ROC Curves depicting the performance of Random Forest (RF), eXtreme Gradient Boosting (XGBoost), and Gradient Boosting Machine (GBM) algorithms in SHCC (Shanghai Cancer Center) (**a**) and HSH (Huashan Hospital) (**b**). The Area Under the Curve (AUC) values are specified for each model. (**c**,**d**) Performance metrics comparison of the RF, XGBoost, and GBM algorithms, encompassing accuracy, AUC, F1 score, Kappa statistics, precision, sensitivity, and specificity in both SHCC (**c**) and HSH (**d**). (**e**,**f**) Feature importance plots for RF, XGBoost, and GBM algorithms, demonstrating the relative significance of different predictors, with LVI consistently emerging as a paramount predictor for LNM in both SHCC (**e**) and HSH (**f**).

To discern the prognostic significance of LVI in predicting LNM within EGC patients, we adopted an expansive machine learning methodology, exploiting three advanced algorithms: RF, GBM, and XGBoost [16,17,18], as depicted in Figure 2. These algorithms rendered distinguishable predictive performances, which were notably delineated by the AUC among other pivotal metrics, corroborating their predictive viability in the given context. In SHCC, the AUCs achieved were as follows: RF—0.916, XGBoost—0.918, and GBM—0.945 (Figure 2a). Conversely, in HSH, the AUCs were notably lower, with RF rendering 0.823, XGBoost—0.781, and GBM—0.882 (Figure 2b). Despite variances, all models showcased substantive predictive capacities, illuminating an array of crucial metrics including accuracy, sensitivity (recall), specificity, precision, F1 score, and Kappa, each affording distinct perspectives into the model’s operational efficacy and dependability across different contexts (Figure 2c,d).

In examining feature importance, LVI steadfastly emerged as a cardinal predictor for LNM in both SHCC and HSH, thereby accentuating its imperative role in steering predictive modeling within this medical domain (Figure 2e,f). The congruence of this finding across multiple models and datasets accentuates the potential ubiquity of LVI as a prognostic factor within EGC prognostications.

### 3.3. Postoperative Morbidity and Mortality

The overall postoperative complication rate was observed in 16.4% of the cohort, with LVI-positive patients exhibiting a higher complication rate of 23.0%, compared to 15.9% in LVI-negative patients; however, this was not statistically significant (*p* = 0.074) (Table 2). The most common surgical-related complications were postoperative bleeding (2.6%), anastomotic leakage (1.8%), and wound problems (1.6%). Non-surgical-related complications such as pleural effusion (2.2%) and pulmonary infection (1.2%) were also noted. The operative mortality rate was 0.2% in the LVI-negative group, with no mortality reported in the LVI-positive group.

### 3.4. Prognostic Factors in EGC Patients Meeting ESD Criteria at Both Centers

**Figure 3:**
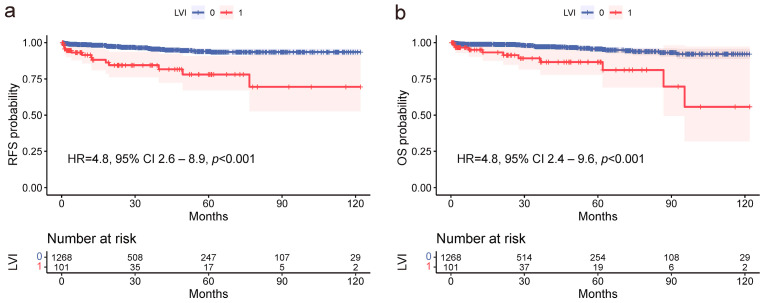
Kaplan–Meier survival analyses elucidating the profound prognostic influence of lymphovascular invasion (LVI) on recurrence-free survival (RFS) and overall survival (OS) amongst ESD-eligible EGC patients. (**a**) LVI is identified as a significant predictor of reduced RFS (HR = 4.8, 95% CI 2.6–8.9, *p* < 0.001). (**b**) LVI is associated with a significant reduction in OS (HR = 4.8, 95% CI 2.4–9.6, *p* < 0.001).

The median follow-up period of patients was 68 months (range 24–120 months). The 5-year survival rate was 95.7% in the LVI-negative group and 86.6% in the LVI-positive group. Univariate analysis showed that age ≥ 70, presence of LNM, and LVI were significant negative predictors for RFS, with HRs of 3.6 (95% CI 2.0–6.4, *p* < 0.001), 6.2 (95% CI 3.2–12.2, *p* < 0.001), and 4.6 (95% CI 2.4–8.6, *p* < 0.001), respectively (Table 3, Figure 3a). In the multivariate analysis, age and LVI remained significant predictors, with HRs of 3.6 (95% CI 2.0–6.4, *p* < 0.001) and 4.5 (95% CI 2.4–8.5, *p* < 0.001), respectively. Similarly, the same factors were significant in the univariate analysis for OS, with HRs of 3.0 (95% CI 1.5–5.9, *p* < 0.001) for age, 5.1 (95% CI 2.4–11.2, *p* < 0.001) for LNM, and 4.5 (95% CI 2.2–9.3, *p* < 0.001) for LVI (Table 3, Figure 3b). Upon multivariate analysis, age and LVI sustained significance, with HRs of 2.9 (95% CI 1.5–5.8, *p* = 0.002) and 4.4 (95% CI 2.1–8.9, *p* < 0.001), respectively. Overall, LVI emerged as a significant prognostic factor for both RFS and OS, underscoring the crucial role of LVI in the prognosis of ESD-eligible EGC patients.

## 4. Discussion

The relationship between LVI and survival metrics in ESD-eligible EGC patients has hitherto been subject to debate. Our multicenter research underscores a substantial correlation between LVI and LNM in this demographic. Moreover, LVI emerges as a salient independent predictor for both RFS and OS among EGC patients adhering to ESD criteria. Consequently, despite the existing management guidelines, this study advocates for a critical reappraisal of treatment protocols for ESD-qualified, LVI-positive EGC patients.

LVI is generally regarded as a preliminary step towards LNM and subsequent micrometastasis [8]. Several studies suggest a close correlation between LVI and a significant increase in LNM in submucosal cancer patients, designating LVI as a crucial risk factor for LNM even in mucosal cancer cases [19]. However, this assertion has been challenged by some studies that do not attribute significant importance to LVI in LNM [20,21]. Such discrepancies might arise due to the low detection rate of LVI or potential confounding factors during statistical analysis. Our study confirms a strong correlation between LVI and LNM among EGC patients. The LNM rates stood at 4.3% among all EGC patients, which significantly increased to 43.6% in patients who are LVI-positive. For EGC patients meeting ESD criteria, LVI significantly influenced the LNM rates. The evidence derived from gastric cancer radical surgery specimens strengthens the understanding of LVI’s pivotal role in LNM.

Our study also reiterates the well-established adverse prognostic role of LVI in EGC. We found that LVI independently predicts inferior RFS and OS among EGC patients meeting ESD criteria. These results align with previous studies highlighting the negative prognostic impact of LVI in gastric cancer [12]. Indeed, several studies have demonstrated that LVI status significantly influences 5-year disease-specific survival rates in ESD-treated mucosal gastric cancer [22]. This suggests that additional surgical resection may be necessary in LVI-positive cases.

Our study incorporates data from SHCC and HSH, two centers with distinct patient intake capacities, potentially affecting the spectrum of patient cases encountered. This discrepancy leads to notable variations in essential clinical and pathological characteristics, such as age, tumor location, tumor grade, and the presence of ulcers. Despite these variations in patient profiles between SHCC and HSH, the impact of LVI as a consistent prognostic predictor is a significant finding. This underscores the clinical importance of LVI, highlighting its role as a robust prognostic marker that transcends patient cohort differences at these two institutions. The uniformity of our findings across these diverse clinical settings enhances the generalizability and applicability of our conclusions.

While current guidelines recognize the significance of LVI in EGC treatment post-ESD, they tend to offer more general rather than explicit recommendations for managing EGC patients with LVI after ESD. This leaves a degree of interpretation in clinical decision making, particularly concerning the use of additional surgery or intensive follow-up. Our research supplements the expanding compendium of evidence supporting the detrimental prognostic implications of LVI, even among EGC patients who meet standard criteria for ESD. Thus, we propose a cautious re-evaluation of management strategies for LVI-positive EGC eligible for ESD. Coupling the TNM staging system with independent prognostic indicators such as LVI could potentially yield a more nuanced and precise assessment of patient prognosis and facilitate improved prognostic stratification for distinct subgroups [23].

In our investigation, a significant subset of patients, despite being eligible for ESD, underwent radical gastrectomy. This trend reflects the evolving landscape of gastric cancer management in China, where ESD’s integration into clinical practice is relatively recent, especially when compared to its earlier adoption in Japan and Korea [24,25]. The gradual embracement of ESD by the Chinese medical community is a progressive yet transitional phase, shaped by factors such as ESD’s innovation status, the current clinical guidelines, and the dynamics of decision making between patients and physicians. ESD, characterized by its minimally invasive nature and quicker recovery times, has raised concerns regarding the adequacy of lymph node metastasis assessment and uncertainties about its long-term outcomes [26]. Consequently, radical gastrectomy, which is recognized for its comprehensive approach to managing lymph node metastasis, is often favored in situations where the risk of lymph node involvement is not clearly defined. This inclination towards radical gastrectomy may also signify a cautious approach in the adoption of newer medical techniques within the Chinese healthcare setting. This pattern of treatment choices underscores a pivotal aspect of medical practice in China—the intricate balance between embracing cutting-edge, minimally invasive methods and relying on established, traditional surgical techniques. It highlights the necessity for ongoing education and training, along with the thorough evaluation of emerging treatment modalities. Ensuring their effective and safe implementation into clinical practice is essential, particularly in a context that takes into account the specificities of regional healthcare systems and aligns with patient preferences and expectations.

Radical surgery may serve to reduce recurrence rates by clearing residual tumor and lymph nodes, thus potentially mitigating the adverse prognosis associated with LVI. However, patients who opted not to undergo further surgical intervention following ESD displayed significantly poorer outcomes in comparison to their counterparts who underwent additional surgery [27]. Corroborating this finding, a retrospective study comparing EGC cases extending beyond the scope of endoscopic resection showed negligible variance in both mortality and recurrence rates of gastric cancer between patients who initially opted for gastrectomy and those who pursued it post-ESD [28]. In contrast, those who abstained from gastrectomy after ESD manifested significantly higher rates, underscoring the potential benefits of additional surgical intervention [29]. Our study highlights that EGC patients meeting ESD criteria but diagnosed with positive LVI face a less favorable prognosis post-radical surgery than those without LVI. This finding emphasizes the necessity of additional surgical intervention for LVI-positive individuals following ESD. Moreover, our results prompt a re-evaluation of treatment strategies for patients with comorbidities. Despite the complexities these comorbidities introduce, the heightened risk of LNM in LVI-positive patients underscores the potential advantages of surgical intervention. We advocate for a surgical-centric, personalized approach, carefully weighing cancer-specific risks against the overall health profile of the patient to enhance outcomes.

Certain limitations of this study deserve acknowledgment. Given its retrospective nature, the potential for patient selection bias cannot be overlooked. However, we believe that the broad, multicentric scope of our research may offset such bias, ensuring a diverse and representative sample. Regarding the identification of LVI, our study utilized standard Hematoxylin and Eosin (H&E) staining. While it is acknowledged that implementing immunohistochemical staining could potentially enhance the sensitivity and specificity of LVI detection, our research did not routinely apply this method [30,31]. Nonetheless, the LVI incidence in this study aligns with rates reported in previous studies. Therefore, we posit that the clinical relevance of LVI detection remains substantial, even when relying solely on conventional H&E staining without the support of immunohistochemical procedures. Finally, LVI status was determined based on postoperative histopathology. Future prospective studies are needed to validate risk stratification based on LVI and investigate optimal management strategies for EGC patients with this poor prognostic feature.

## 5. Conclusions

Our study highlights the significant role of LVI in guiding treatment decisions for EGC patients eligible for ESD. The detection of LVI in gastric biopsy advocates for radical gastrectomy as a superior option due to its effectiveness in mitigating the increased risk of regional and metastatic spread. This is particularly critical for patients diagnosed with LVI post-ESD, where additional surgical intervention is imperative for complete tumor resection and the effective management of metastatic pathways. Furthermore, this study underscores the complexity of managing EGC patients with comorbidities, emphasizing the necessity of integrating LVI status into treatment planning to refine risk stratification and tailor postoperative management. Understanding the significance of LVI aids clinicians in discussing treatment options with patients, enabling more informed decision making and personalized patient care. Additional investigation is also warranted to evaluate the potential utility of incorporating LVI status into existing TNM staging systems and to explore novel therapeutic targets and personalized treatment strategies.

## Figures and Tables

**Table 1:** Relationship between clinicopathological factors and lymphovascular invasion (LVI) in EGC meeting ESD criteria.

	Total (N)	LVI-Positive	LVI-Negative	*p* Value
Mean age (years)	58.5 ± 10.8	58.0 ± 12.0	58.6 ± 10.7	0.629
Sex				
M	934 (68.2)	50 (49.5)	884 (69.7)	<0.001
F	435 (31.8)	51 (50.5)	384 (30.3)	
Tumor location				
U	197 (14.4)	8 (7.9)	189 (14.9)	0.081
M	226 (16.5)	22 (21.8)	204 (16.1)	
L	946 (69.1)	71 (70.3)	875 (69.0)	
Differentiation				
Differentiated	932 (68.1)	61 (60.4)	871 (68.7)	0.085
Undifferntiated	437 (31.9)	40 (39.6)	397 (31.3)	
Depth				
M	1060 (77.4)	62 (61.4)	998 (78.7)	<0.001
SM	309 (22.6)	39 (38.6)	270 (21.3)	
Tumor size				
≤10 mm	476 (34.8)	25 (24.8)	451 (35.6)	0.071
≤20 mm	628 (45.9)	51 (50.5)	577 (45.5)	
>20 mm	265 (19.4)	25 (24.8)	240 (18.9)	
Ulcer finding				
Absence	1259 (92.0)	96 (95.0)	1163 (91.7)	0.236
Presence	110 (8.0)	5 (5.0)	105 (8.3)	
Nerve invasion				
Absence	1358 (99.2)	96 (95.0)	1262 (99.5)	<0.001
Presence	11 (0.8)	5 (5.0)	6 (0.5)	
Lymph node metastasis				
Absence	1310 (95.7)	57 (56.4)	1253 (98.8)	<0.001
Presence	59 (4.3)	44 (43.6)	15 (1.2)	
N stage				
N0	1040 (92.9)	36 (37.5)	1004 (98.1)	<0.001
N1	55 (4.9)	41 (42.7)	14 (1.4)	
N2	18 (1.6)	15 (15.6)	3 (0.3)	
N3	6 (0.5)	4 (4.2)	2 (0.2)	
Dissection				
D1/D1+	673 (49.2)	38 (37.6)	633 (49.9)	0.017
D2	696 (50.8)	63 (62.4)	635 (50.1)	
Reconstruction				
Billroth-I	433 (31.6)	29 (28.7)	404 (31.9)	0.795
Billroth-II	138 (10.1)	11 (10.9)	127 (10.0)	
Billroth-II with Braun	273 (19.9)	26 (25.7)	247 (19.5)	
Roux-en-Y after distal gastrectomy	198 (14.5)	15 (14.9)	183 (14.4)	
Roux-en-Y after total gastrectomy	177 (12.9)	10 (9.9)	167 (13.2)	
Esophagogastrostomy after proximal gastrectomy	103 (7.5)	7 (6.9)	96 (7.6)	
Double-tract after proximal gastrectomy	47 (3.4)	3 (3.0)	44 (3.5)	

**Table 2:** Postoperative morbidity and mortality.

	Total (%)	LVI-Positive	LVI-Negative	*p* Value
Overall complication	225 (16.4)	23 (23.0)	202 (15.9)	0.074
Surgical-related				
Wound problem	22 (1.6)	2 (2.0)	20 (1.6)	0.757
Postoperative bleeding	35 (2.6)	3 (3.0)	32 (2.5)	0.784
Anastomotic leakage	25 (1.8)	2 (2.0)	23 (1.8)	0.904
Abdominal infection	16 (1.2)	1 (1.0)	15 (1.2)	0.862
Intestinal obstruction	4 (0.3)	0 (0)	4 (0.3)	0.572
Stenosis	6 (0.4)	1 (1.0)	5 (0.4)	0.383
Gastric stasis	7 (0.5)	1 (1.0)	6 (0.4)	0.483
Lymphatic leakage	10 (0.7)	1 (1.0)	9 (0.7)	0.750
Non-surgical-related				
Pulmonary infection	17 (1.2)	1 (1.0)	16 (1.3)	0.812
Pleural effusion	31 (2.2)	4 (4.0)	27 (2.1)	0.234
Cardiovascular system	9 (0.7)	1 (1.0)	8 (0.6)	0.667
Cerebrovascular disease	5 (0.4)	0 (0)	5 (0.4)	0.527
Urinary problem	7 (0.5)	1 (1.0)	6 (0.4)	0.488
Hepatic problem	10 (0.7)	2 (2.0)	8 (0.6)	0.128
Others	21 (1.5)	3 (3.0)	18 (1.4)	0.223
Operative mortablity	2 (0.1)	0 (0)	2 (0.2)	0.69

**Table 3:** Factors associated with RFS and OS.

	RFS				OS			
	Univariate Analysis		Multivariate Analysis		Univariate Analysis		Multivariate Analysis	
	HR (95% CI)	*p* Value	HR (95% CI)	*p* Value	HR (95% CI)	*p* Value	HR (95% CI)	*p* Value
Age								
≥70 vs. <70	3.6 (2.0–6.4)	<0.001	3.4 (1.9–6.1)	<0.001	3.0 (1.5–5.9)	<0.001	2.9 (1.5–5.8)	0.002
Sex								
M vs. F	1.2 (0.7–2.2)	0.485			1.2 (0.6–2.4)	0.517		
Tumor location								
M vs. U	1.0 (0.5–2.1)	0.991			1.4 (0.6–3.1)	0.416		
L vs. U	0.8 (0.4–1.7)	0.559			0.9 (0.4–2.1)	0.821		
Differentiation								
Undifferntiated vs. Differentiated	0.6 (0.3–1.1)	0.126			0.6 (0.3–1.2)	0.163		
Depth								
SM1 vs. M	1.0 (0.5–2.0)	0.931			0.9 (0.4–1.9)	0.720		
Tumor size								
>20 mm vs. ≤20 mm	1.5 (0.8–2.9)	0.221			1.8 (0.9–3.7)	0.106		
Ulcer finding								
Presence vs. Absence	1.3 (0.6–3.0)	0.535			1.6 (0.7–3.7)	0.313		
Lymph node metastasis								
Presence vs. Absence	6.2 (3.2–12.2)	<0.001	——	——	5.2 (2.4–11.2)	<0.001	——	——
Lymphovascular invasion								
Presence vs. Absence	4.8 (2.6–8.9)	<0.001	4.5 (2.4–8.6)	<0.001	4.8 (2.4–9.6)	<0.001	4.7 (2.3–9.4)	<0.001
Nerve Invasion								
Presence vs. Absence	4.1 (1.0–16.9)	0.050			2.7 (0.4–19.5)	0.331		

## Data Availability

The datasets generated and/or analyzed during the current study are available from the corresponding author upon reasonable request.
